# Students experience the effects of climate change on children’s health in role play and develop strategies for medical work – an interactive seminar

**DOI:** 10.3205/zma001611

**Published:** 2023-05-15

**Authors:** Friederike Jonas, Anja Hagen, Benjamin W. Ackermann, Matthias Knüpfer

**Affiliations:** 1 Universitätsklinikum Leipzig, Klinik und Poliklinik für Kinder- und Jugendmedizin, Leipzig, Germany; 2 Universitätsklinikum Leipzig, Klinik und Poliklinik für Kinder- und Jugendmedizin, Selbstständige Abteilung für Neonatologie, Leipzig, Germany

**Keywords:** medical education, climate change, global warming, global health, pediatrics, perinatology

## Abstract

**Background:**

This project report describes the development and evaluation of an interactive seminar on the topic “medical effects of climate change on children's health”.

**Objectives:**

The learning objectives are learning the basics and the direct and indirect connections between climate change and children's health. Future scenarios for affected children, parents and doctors are developed interactively. Subsequently, communication strategies concerning climate change are discussed so that students identify and analyze possibilities to become active.

**Methodology:**

The seminar was offered as an obligatory seminar for a total of 128 third-year medical students with one appointment of 45 minutes per course group as part of the interdisciplinary seminar series “Environmental Medicine”. A course group consisted of 14 to 18 students. The seminar for the 2020 summer semester was developed as part of the interdisciplinary field of environmental medicine with the special feature of an interactive role play. The role play intends to give the students the opportunity to put themselves in the situation of affected children, parents and doctors of the future in order to develop detailed solution strategies. From 2020 to 2021, the seminar took place as online self-study due to the lockdown requirements. Since winter semester 2021/22, the seminar was held as an attendance event for the first time, although the switch to an online presence seminar with obligatory attendance had to take place after four seminar dates due to renewed lockdown requirements, which also took place four times. The evaluated results here refer to a total of eight dates in the winter semester 2021/22 and were carried out using a specially developed questionnaire, which was filled out voluntarily and anonymously by the students immediately after the respective seminar date. An overall grade as well as the appropriateness of the time and content of lectures and role play were asked for. Free text answers were possible for each question.

**Results:**

A total of 83 questionnaires were evaluated, 54 of which were from the four seminars in attendance, 15 were from the four online presence seminars that took place as an online live stream. The evaluation of the seminar resulted in an average grade of 1.7 for the face-to-face seminars and 1.9 for the online seminars. Content-related comments in the free-text answers addressed the desire for concrete solution strategies, more time for discussions and a more in-depth study of the topic. Numerous positive responses described the seminar as “very exciting”, “good food for thought”, “interesting and important topic”.

**Conclusion:**

There is a very high interest on the topic of “climate change & health” among students There is an obvious need to integrate the topic on a larger scale into medical education. Ideally, the focus on children's health should be an integral part of the pediatric curriculum.

## Zusammenfassung

**Hintergrund:**

In diesem Projektbericht wird die Erarbeitung und Auswertung eines interaktiven Seminars mit dem Thema „Medizinische Auswirkungen des Klimawandels auf die Kindergesundheit“ beschrieben.

**Zielsetzung:**

Die Lernziele sind das Erlernen der Grundlagen sowie der direkten und indirekten Zusammenhänge zwischen Klimawandel und Kindergesundheit. Interaktiv werden Zukunftsszenarien betroffener Kinder, Eltern und Mediziner*innen erarbeitet. Im Anschluss werden Kommunikationsstrategien über den Klimawandel besprochen, sodass die Studierenden die Möglichkeiten aktiv zu werden identifizieren und analysieren.

**Methodik:**

Das Seminar wurde als Pflichtseminar für insgesamt 128 Humanmedizin-Studierende im dritten Studienjahr mit einem Termin á 45 Minuten pro Kursgruppe im Rahmen der fächerübergreifenden Seminarreihe „Umweltmedizin“ angeboten. Eine Kursgruppe bestand aus 14 bis 18 Studierenden. Entwickelt wurde das Seminar für das Sommersemester 2020 im Rahmen des Querschnittbereich Umweltmedizin mit der Besonderheit eines interaktiven Rollenspiels. Das Rollenspiel soll den Studierenden die Möglichkeit geben, sich in die Situation von betroffenen Kindern, Eltern und Mediziner*innen der Zukunft zu versetzen, um konkrete Lösungsstrategien zu erarbeiten. Von 2020 bis 2021 erfolgte das Seminar auf Grund der Lockdown-Vorgaben als Online-Eigenstudium. Seit dem Wintersemester 2021/22 wird das Seminar erstmals als Präsenz-Seminar durchgeführt, wobei nach vier Seminarterminen wegen erneuter Lockdown-Vorgaben die Umstellung auf ein Online-Live-Seminar mit Anwesenheitspflichtstattfinden musste, welches ebenfalls viermal stattfand. Die hier ausgewertete Evaluation bezieht sich auf die insgesamt acht Termine im WS 2021/22 und erfolgte anhand eines eigens entwickelten Fragebogens, welcher unmittelbar nach dem jeweiligen Seminartermin von den Studierenden freiwillig und anonym ausgefüllt wurde. Hier wurde nach einer Gesamtnote sowie Angemessenheit des zeitlichen und inhaltlichen Umfangs von Vorträgen und Rollenspiel gefragt. Freitextantworten waren zu jeder Frage möglich.

**Ergebnisse:**

Es wurden insgesamt 83 Fragebögen ausgewertet, 54 davon lagen aus den vier Seminaren in Präsenz vor, 15 lagen aus den vier Seminaren vor, welche als Online-Livestream stattgefunden haben. Die Evaluation des Seminars ergab eine Durchschnittsnote von 1,7 als Mittelwert für die Präsenz-Seminare und von 1,9 für die Online-Seminare. Inhaltliche Bemerkungen in den Freitextantworten thematisierten den Wunsch nach konkreten Lösungsstrategien, mehr Zeit für Diskussionen und einer inhaltlichen Vertiefung des Themas. Zahlreiche positive Rückmeldungen beschrieben das Seminar als „sehr spannend“, „guter Denkanstoß“, „interessantes und wichtiges Thema“.

**Schlussfolgerung:**

Das Interesse der Studierenden am Thema „Klimawandel & Gesundheit“ ist sehr groß. Es zeigt sich die deutliche Notwendigkeit, das Thema breiter in die medizinische Ausbildung zu integrieren. Der Fokus auf die Gesundheit der Kinder sollte bestenfalls fester Bestandteil des pädiatrischen Curriculums sein.

## Introduction

A key goal in the fight against climate change is transferring and imparting knowledge about health consequences of global warming for humanity [1]. In the future, newborns, children and adolescents will be significantly affected by climate change and will experience effects on their health [2], [3]. These effects will play an important role in the day-to-day work of future physicians. The Alliance for Climate Change and Health has therefore formulated the goal of integrating courses on preventive measures and the health consequences of climate change into student training [4]. As members of the Leipzig local group health for future, which was founded in 2019 through an appeal by the alliance for climate change and health, we have developed an interactive seminar with the main topic “pediatric environmental medicine”.

It was important to the developers of the seminar to address different perspectives on climate change. In addition to the health consequences for children and adults, the health system as a greenhouse gas producer and thus a contributor to the progression of climate change is also discussed. Different points of view should be taken up by the students themselves interactively in role plays. The challenge here is to transfer the previously learned general and theoretical knowledge into concrete practical situations. By assuming different roles of affected parents, young people or their therapists, options and motivation for action can be better understood by empathizing with the situation and strategies for dealing with and solving the problem can be developed [5]. At best, this leads to an intensive examination of the core topic and can increase the intrinsic motivation to take action against climate change [6].

We have been conducting this mandatory seminar since the summer semester 2020 as part of the interdisciplinary subject “environmental medicine” with a course duration of 45 minutes per participant group at the University of Leipzig.

### Learning objectives

The learning objectives of the seminar are:

1. The third-year students know the basics of man-made climate change as well as direct and indirect connections between climate change and children's health.
2. In a role play, the students work out the effects of climate change on the health of affected children and their parents currently and in the future by adopting different perspectives.
3. The students can identify and analyze own opportunities to become active as physicians.

## Material and methodology

**Figure 1:**
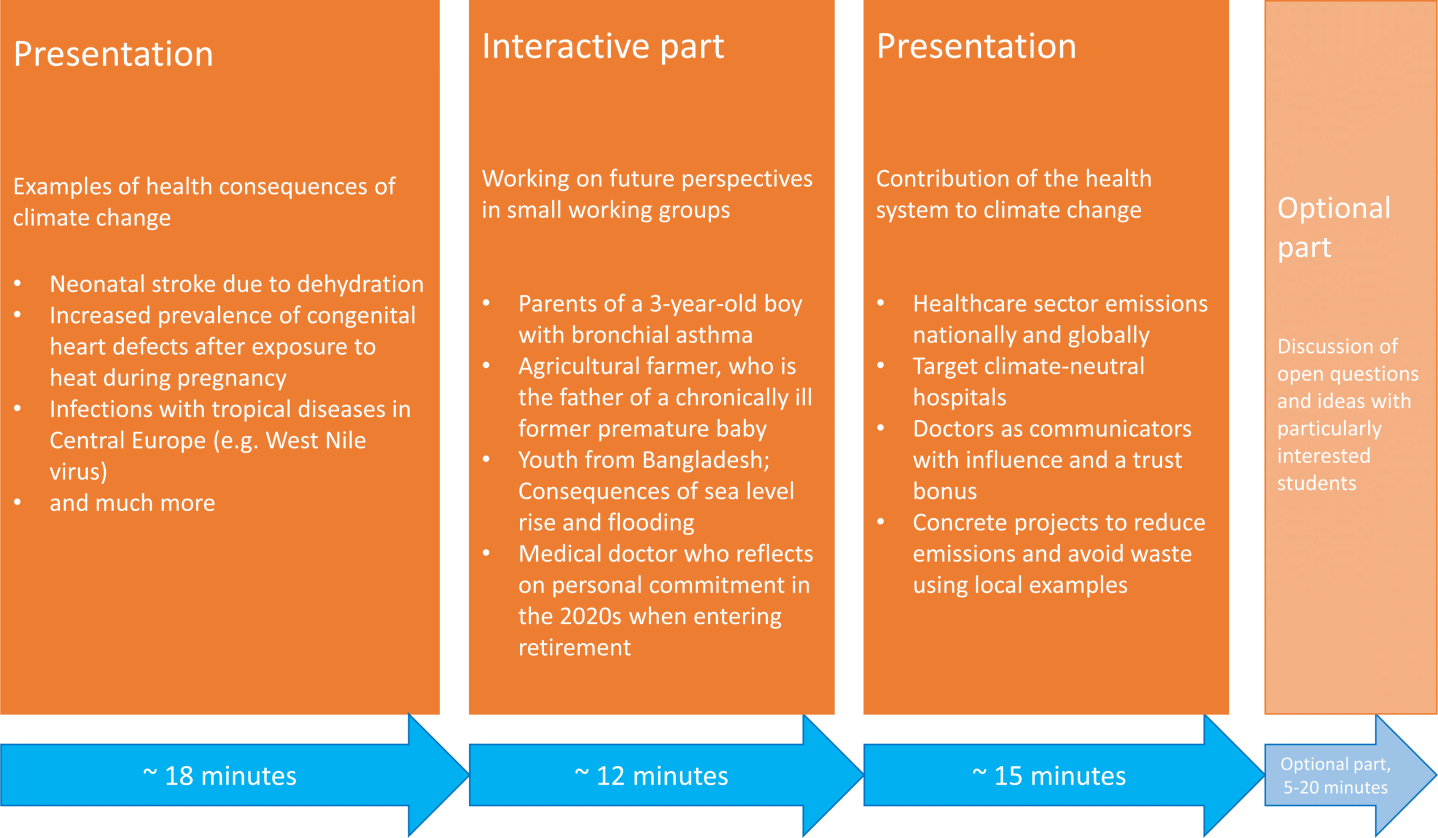
Classification and structure of the seminar “pediatric environmental medicine” in the interdisciplinary seminar series of the interdisciplinary area “environmental medicine” with content and time structure in 3 parts.

**Figure 2:**
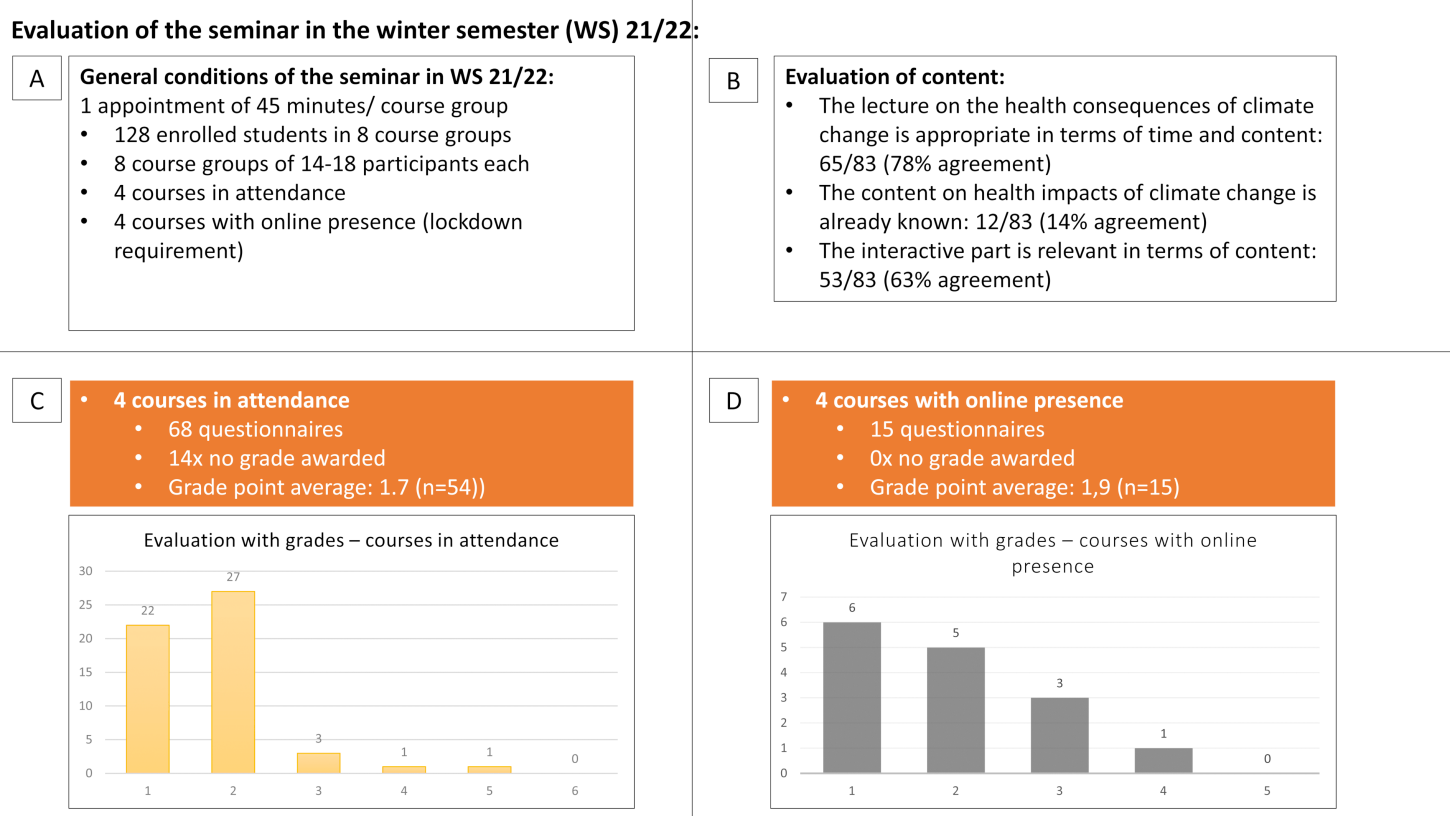
Presentation of the evaluation for the seminar: “pediatric environmental medicine” based on the evaluation of the students from the winter semester 2021/22. A: Summary of the general conditions of the seminar. B: Key statements on the content-related questions of the evaluation. C: Evaluation of the seminar with grades as a course with attendance (orange). D: Evaluation with grades for the seminar as a course with online-presence (grey). The grades are listed on the x-axis and the number of ratings on the y-axis. For each note, the number of notes in question is shown above the bar.

The seminar was developed as a mandatory seminar with an appointment of 45 minutes each as part of the interdisciplinary cross-sectional area of environmental medicine with the special feature of an interactive role play in the summer semester 2020. From 2020 to 2021 the seminar took place as online self-study. Since the winter semester 2021/22, the seminar has been held in presence for the first time, whereby after 4 seminars with physical presence, a course with online live stream with obligatory attendance for another 4 groups took place, due to the lockdown requirements, In the 2021/22 winter semester, a total of 128 students were enrolled in 8 course groups. A course group consisted of 14 to 18 students. The procedure and the general conditions of the seminar are summarized in figure 1 (Fig. 1) and figure 2A (Fig. 2).

The seminar was evaluated immediately after each of the 4 face-to-face sessions using a printed questionnaire, which could be filled out individually on a voluntary and anonymous basis. After the 4 online appointments, the participants were given a link to an online questionnaire for evaluation. In the evaluation, yes/no questions and free text questions were used to ask for a grade, for a subjective assessment of the scope of time and content, and for the respective relevance of the 3 seminar parts. Free text answers are possible for each question. The evaluation form used for the face-to-face course is shown in attachment 1 , the evaluation used in the online course corresponded to the printed form. Since different tools were used for the evaluations, grades 1 to 6 could be selected for the face-to-face courses and grades 1 to 5 for the online courses.

## Project description

The 45-minute seminar is divided into three parts according to the learning objectives mentioned above, which are outlined in figure 1 (Fig. 1).

**Figure 3:**
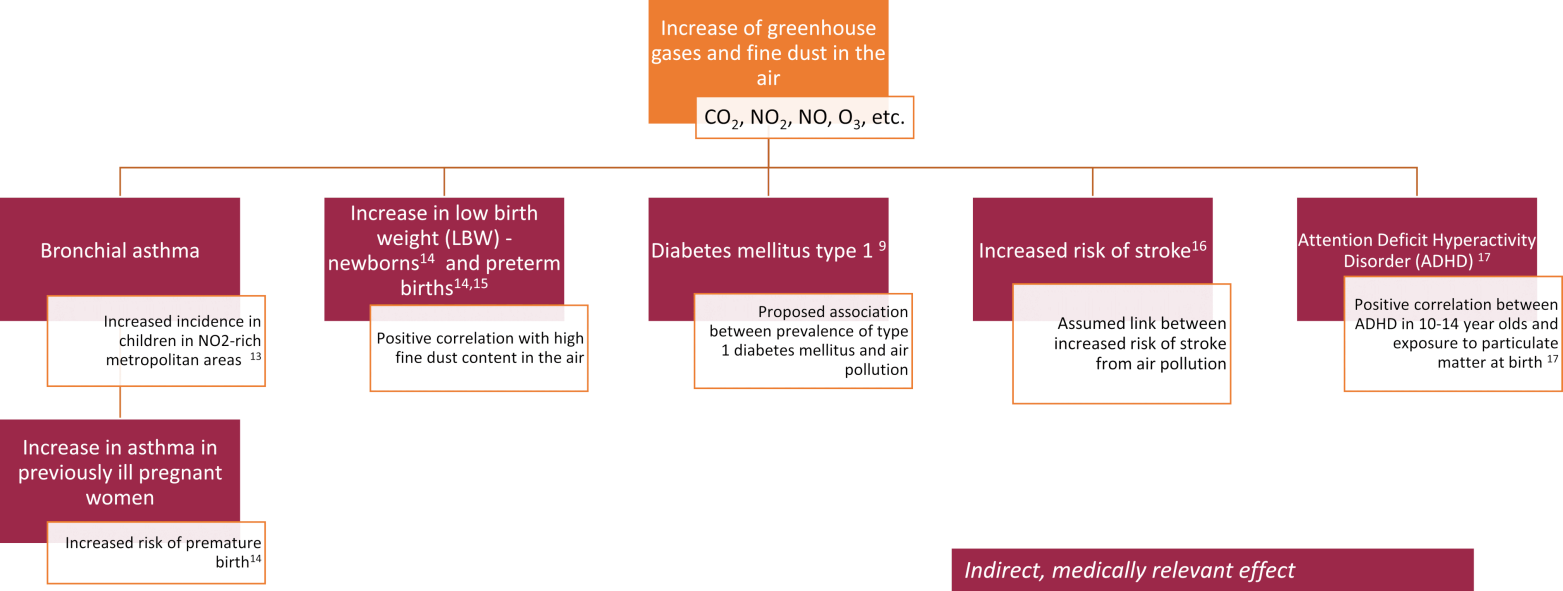
Possible health effects (in red) of increased air pollution, e.g. with carbon dioxide, nitrogen dioxide, nitrogen monoxide and ozone (in orange). Indirect, medically relevant consequences for vulnerable groups such as pregnant women, newborns, children and young people are shown, as they are significantly more affected by the consequences of climate change.

The first part consists of a lecture on the health consequences of climate change, which in terms of content particularly describes the consequences for newborns, children and young people. These are direct consequences that are easy to understand, but also yet unexplained links. The understandable consequences include, for example, a higher prevalence of dehydrated children during heat and drought periods or an increase in accidents and falls during extreme weather events [7]. The still unexplained phenomena include, for example, an increased prevalence of heart defects in fetuses whose mothers experienced a high proportion of hot days in the first trimester [8] or, for example, the ever earlier onset of type 1 diabetes mellitus in connection with higher air pollution [9]. The health effects of increased air pollution are shown in figure 3 (Fig. 3) as an example. Many of the health consequences are of concern to everyone, but vulnerable groups such as pregnant women, newborns, children and young people are hit much more severely 

In the second part, an interactive role play is carried out with a final presentation of the results by the individual groups in the plenary session. In four small groups with a maximum of five people per group, the students take a future perspective with the aim of working out the consequences of global warming for children and young people. By means of interactive role play, the students should be able to feel empathy for those affected, which explains the relevance of the effects on the health of the individual as well as the necessity and competence of the medical need for action. The following three roles are specified here:

1. Parents of a three-year-old asthma patient who grows up in a city polluted with fine dust.
2. A farmer who takes care of his family with three children, one of them a chronically ill former premature baby.
3. A youth living in Bangladesh with his extended family who is suffering the devastating effects of a flood.

The effects for those affected should be able to be concluded from the information from the first day before. As additional information, the students receive current data on fine dust pollution in Stuttgart (role 1), on the water balance in Saxony (role 2) and on the loss of land on the coast of Bangladesh (role 3). There is also space for creative content, which can be discussed in the plenary session after presentation by the small group.

**Figure 4:**
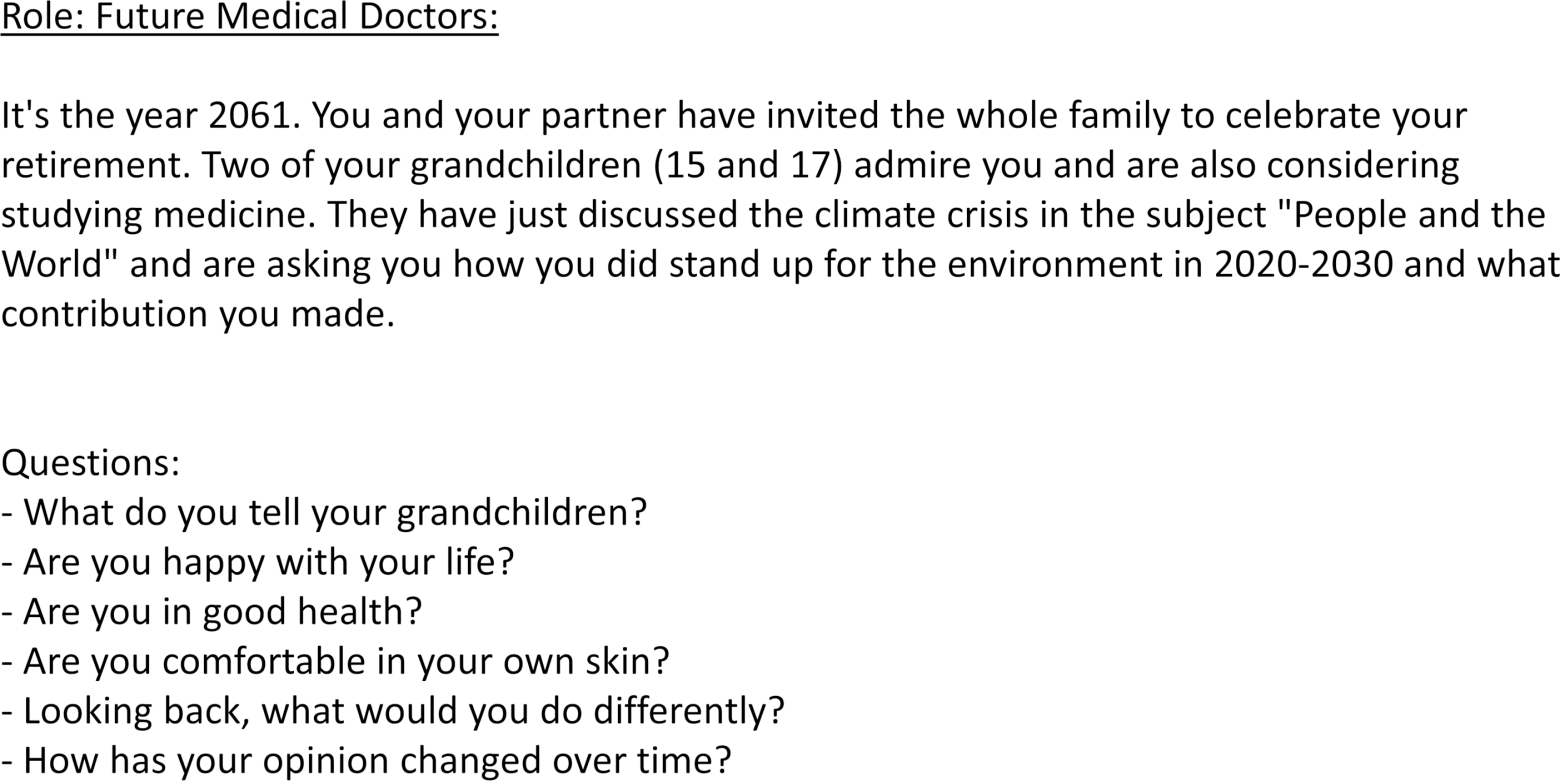
This part of the interactive role-play has the goal of developing solution strategies for the health professions. It is intended to serve as a transition between the role of affected parents or children and the role of health professionals. The aim is to put oneself in the perspective of medical doctors of the future who have already been able to help limit global warming.

With the aim of developing solution strategies, another role is to put oneself in the perspective of doctors in the future who have already been able to contribute to limiting global warming. This role is shown in figure 4 (Fig. 4) as an example. 10 minutes are intended for the group work, and 3 minutes per group for the presentation of the results in the plenary session.

In the third part of the seminar, the contribution of the health system to climate change and the role of students and doctors in the fight against climate change will be presented. In detail, it is about the emissions caused by our healthcare system and the urgent need for climate-neutral hospitals. Furthermore, the trust of the population in doctors and health care workers is discussed and how a personal level of communication about climate change can be achieved. The listeners are thus encouraged to position themselves as future doctors and to become active.

## Results

128 enrolled students from the 3rd academic year of the 2021/21 winter semester had the opportunity to evaluate. A total of 83 questionnaires were available for evaluation, 68 of them from four face-to-face seminars and 15 from four live online seminars. 14 out of 83 participants did not give a grade. The evaluation of the seminar resulted in an average grade of 1.7±0.81 as the mean ± standard deviation from all grades awarded (N=54) for the four face-to-face seminars and an average grade of 1.9±0.93 as the mean ± standard deviation of all grades awarded (N=15) for two out of five online seminars. The evaluation of the seminar is summarized in figure 2 (Fig. 2), the evaluation of content in figure 2B (Fig. 2) and the distribution of the grades in figure 2C/D (Fig. 2).

The further analysis of the evaluation describes the proportion of participants who agreed with the following statements. In 65 of 83 (78%) of the assessments, the lecture on the health consequences of climate change was rated as appropriate in terms of time and content. Only 12 of 83 (14%) participants were already familiar with the content of this lecture. 53 of 83 (65%) participants found the content of the interactive role play relevant. Free text answers to the role play were, for example: “exciting change of perspective” and “it’s nice to put yourself in the shoes of others”. In the online seminars, clearer instructions for working in the interactive part were requested.

Further content-related feedback in the free-text answers addressed great interest in the topic of climate change and health. One response to this was: “Due to the importance of the topic, it would be great to integrate even more events like this into the course in the future. Thank you!” Furthermore, the desire for concrete solution strategies in the health sector and a deepening of the “climate-friendly hospital” was expressed. In addition to the medical consequences, there was also interest in a detailed presentation of non-medical consequences of climate change.

The wish for more time for discussions was expressed several times. Numerous positive responses described the seminar as “very exciting”, “good food for thought”, “very nice to put yourself in the shoes of others”, “interesting and important topic”. Unfortunately, there were no specific criticisms or requests for improvement from grade groups 3 to 5.

## Discussion

Healthcare workers are currently not sufficiently prepared for the health consequences of climate change [10]. So far, these have not been sufficiently addressed in the curriculum, only a few participants describe the seminar content as already fully known. In medical training, among other things, the diagnosis and therapy of tropical diseases could be given more emphasis. In healthcare facilities, the premises and equipment would have to be adapted to the rising temperatures. The students' free-text answers make it clear how relevant knowledge about the health effects of climate change is for keeping society healthy and thus for medical education. Only once in the seminar was the grade “insufficient” awarded. The free text answers suggest that this is a participant who is very critical of man-made climate change.

After four attendance dates, the seminar was continued as an online live seminar with compulsory attendance due to renewed COVID-19 related restrictions. Since the evaluations show a better acceptance of the seminar, especially the interactive part, in face-to-face form, we are aiming for a face-to-face seminar in the future.

After evaluating the first seminar units, it became clear that most students have a broad scientific knowledge of the origin of climate change and are also aware of anthropogenic causes. Since the curriculum for the unit “pediatric environmental medicine” is only 45 minutes, the part of the basic teaching was shortened to the essential pediatric and perinatal aspects. The time gained should be used for discussion time in the interactive part. Another option for gaining additional discussion time would be to offer the first section as a blended learning offer, which would be made available to the students as an online lecture in preparation for the seminar and the start of the seminar would begin immediately with group work.

In the interactive part, the personal effects of climate change are to be experienced by feeling empathy with affected people in role-playing games. Re-experiencing can, among other things, strengthen the intrinsic motivation and opinion-forming of the students [6]. Only those who are convinced of the serious health effects of climate change will raise the issue in front of their patients.

Since the students' free text answers expressed the desire for concrete solution strategies, we presented communication strategies in climate change discussions during the course of the seminar. One possibility is, for example, to convince the dialog partner of the necessity of action by creating a common personal level through topics such as health, family, home, security, justice [11], [12]. Communication strategies should be clearly conveyed to the students in future seminars.

## Conclusion

Knowledge and knowledge transfer about climate impacts is the basis for transformative action. The evaluations suggest that the students are very interested in the topic of “climate change & health” and that there is a demand for more in-depth study, especially on the health effects. There is a clear need to integrate the topic more broadly and intensively into medical education. The climate crisis is subject to constant change, so constant scientific updating will always be necessary. Students make a very good contribution to the continuous development of the seminar through their evaluations. The focus on children’s health was already addressed in detail by the Lancet countdown on climate change in 2019 and should ideally be an integral part of the pediatric curriculum.

## Author's ORCID

- Benjamin Ackermann:
https://orcid.org/0000-0003-0836-7176

## Funding

This article was funded by the Open Access Publication Fund of the University of Leipzig.

## Competing interests

The authors declare that they have no competing interests. 

## Supplementary Material

Evaluation sheet for the seminar about pediatric environmental medicine
