# Supplementary material for: Students experience the effects of climate change on children’s health in role play and develop strategies for medical work – an interactive seminar
Source: GMS J Med Educ. 2023 May 15;40(3):Doc29. doi: 10.3205/zma001611 (PMC10291342; doi:10.3205/zma001611)
Supplement: Evaluation sheet for the seminar about pediatric environmental medicine [file JME-40-29-s-001.pdf]

Attachment 1: Evaluation sheet for the seminar about pediatric environmental medicine

1. I rate the seminar overall with the grade:
   - ☐ □ Very good
   - ☐ □ Good
   - ☐ □ Satisfactory
   - ☐ □ Sufficient
   - ☐ □ Poor
   - ☐ □ Insufficient

2. In the following, please evaluate the first part of the seminar (first presentation on the health consequences of climate change).
   I agree to the following statement(s):
   - I found the time and content of the first presentation to be appropriate □ yes □ no
   - The content of the first presentation was already known to me □ yes □ no
   - The content of the first presentation was not relevant to me □ yes □ no

   Further comments on the first part of the seminar, possibly justification for the above answers:
   ____

3. Please evaluate the second part of the seminar below (interactive role play).
   I agree to the following statement(s):
   - I found the interactive part entertaining □ yes □ no
   - I found the interactive part relevant in terms of content □ yes □ no
   - I found the interactive role-play to be appropriate in terms of time □ yes □ no

   Further comments on the second part of the seminar, possibly justification for the above answers:

   ____

4. Please evaluate the third part of the seminar (second presentation on the contribution of the health sector to climate change).
   I agree to the following statement(s):
   - I found the second presentation to be appropriate □ yes □ no
   - The content of the second presentation was already known to me □ yes □ no
   - I found the second presentation to be appropriate in terms of time □ yes □ no
   - The content of the second presentation was not relevant to me □ yes □ no

   Further comments on the third part of the seminar, possibly justification for the above answers:

   ____

5. Suggestions for improvement/ Feedback:

Attachment 1 to Jonas F, Hagen A, Ackermann BW, Knüpfer M. *Students experience the effects of climate change on children’s health in role play and develop strategies for medical work – an interactive seminar.* GMS J Med Educ. 2023;40(3):Doc29. DOI: 10.3205/zma001611
